# P2X7 receptors in satellite glial cells mediate high functional expression of P2X3 receptors in immature dorsal root ganglion neurons

**DOI:** 10.1186/1744-8069-8-9

**Published:** 2012-02-07

**Authors:** Yong Chen, Guangwen Li, Li-Yen Mae Huang

**Affiliations:** 1 Department of Neuroscience and Cell Biology, University of Texas Medical Branch, 301 University Boulevard, Galveston, TX 77555-1069, USA

**Keywords:** Purinergic signaling, P2X3 receptors, P2X7 receptors, Dorsal root ganglion, P2X7R-P2X3R inhibitory control, Neuron-glia interactions, Nociception, Abnormal pain, Postnatal immature rats, Development

## Abstract

**Background:**

The purinergic P2X3 receptor (P2X3R) expressed in the dorsal root ganglion (DRG) sensory neuron and the P2X7 receptor (P2X7R) expressed in the surrounding satellite glial cell (SGC) are two major receptors participating in neuron-SGC communication in adult DRGs. Activation of P2X7Rs was found to tonically reduce the expression of P2X3Rs in DRGs, thus inhibiting the abnormal pain behaviors in adult rats. P2X receptors are also actively involved in sensory signaling in developing rodents. However, very little is known about the developmental change of P2X7Rs in DRGs and the interaction between P2X7Rs and P2X3Rs in those animals. We therefore examined the expression of P2X3Rs and P2X7Rs in postnatal rats and determined if P2X7R-P2X3R control exists in developing rats.

**Findings:**

We immunostained DRGs of immature rats and found that P2X3Rs were expressed only in neurons and P2X7Rs were expressed only in SGCs. Western blot analyses indicated that P2X3R expression decreased while P2X7R expression increased with the age of rats. Electrophysiological studies showed that the number of DRG neurons responding to the stimulation of the P2XR agonist, α,β-meATP, was higher and the amplitudes of α,β-meATP-induced depolarizations were larger in immature DRG neurons. As a result, P2X3R-mediated flinching responses were much more pronounced in immature rats than those found in adult rats. When we reduced P2X7R expression with P2X7R-siRNA in postnatal and adult rats, P2X3R-mediated flinch responses were greatly enhanced in both rat populations.

**Conclusions:**

These results show that the P2X7R expression increases as rats age. In addition, P2X7Rs in SGCs exert inhibitory control on the P2X3R expression and function in sensory neurons of immature rats, just as observed in adult rats. Regulation of P2X7R expression is likely an effective way to control P2X3R activity and manage pain relief in infants.

## Background

The DRG neuron is the first (primary) neuron in the somatosensory pathway relaying nociceptive (pain), itch and other sensory information from the skin or internal organs to the brain. The cell bodies (somata) of DRG neurons are densely packed in a DRG. Each neuronal soma is tightly wrapped by a layer of SGCs, which are often coupled with one another through gap junctions [1,2]. A neuronal soma with its surrounding SGCs is frequently enclosed by a connective tissue sheath and forms a distinct morphological unit [3]. There is no evidence that classical synaptic contacts exist between neuronal somata in DRGs [2]. We and others have shown that neuronal somata communicate bidirectionally with their surrounding SGCs in DRGs [4,5] and trigeminal ganglia [6]. The communication modulates the activity of somata thus affecting the afferent inputs into the spinal cord [4].

Purinergic ionotropic P2X3Rs and P2X7Rs are ligand-gated ion channels closely associated with the transmission of nociceptive signals in DRGs [5,7-10]. P2X3Rs are expressed mainly in small- and medium-sized adult DRG neurons [5,11] and P2X7Rs are expressed only in SGCs [5,12,13]. We have shown that the P2X3R and P2X7R are the two major purinergic receptors involved in the bidirectional communication between neurons and SGCs [4,5,14]. P2X3Rs have been shown to participate in peripheral pain signaling [15-18] and P2X7Rs are involved in the maturation and release of cytokines [19]. In addition, P2X7R knock-out mice fail to develop hyperalgesia or allodynic pain under inflammation or nerve injury conditions [20]. The evidence has led to the suggestion that P2X7R activation is related to proinflammatory actions in the nociceptive system [21]. It was therefore unexpected when we found that P2X7R activation in SGCs of adult DRGs tonically inhibits the expression of P2X3Rs in neurons [5]. This inhibitory P2X7R-P2X3R control attenuates the activity of P2X3Rs, thus reducing signaling by P2X3R-expressing neurons, most of which are nociceptors, under normal and injured conditions [5,14].

In addition to signaling in adults, purinergic receptors and ATP are found to actively participate in embryonic development in mice and rats [22-25]. P2X3Rs can be seen in day 9.5 mouse embryos (E9.5) [26] and 12.5 day rat embryos [22]. From E14 to postnatal day 1 (P1), P2X3Rs are present in nearly all DRG neurons. The expression of P2X3Rs begins to decline after birth and is expressed in ~73% of P7 and ~45% of P14 to adult mouse DRG neurons. At the developmental stage (E14-P7), most DRG neurons are small in size [23]. After P7, some neurons start to mature into large cells. From P14 to adults, P2X3Rs are found mainly in small diameter DRG neurons which account for ~50% of total adult mouse DRG neurons [23]. P2X3Rs are found in ~ 40% of adult rat DRG neurons [11]. Although P2X3R expression has been found to decrease with age, it is not known if P2X7R expression increases with age and if the regulation between P2X7 and P2X3 exists in developing DRGs.

## Methods

### Animals

Neonatal (3-16 day old) and adult (~250 g) Sprague Dawley rats were used in this study. For *in vitro *experiments, rats were anesthetized with pentobarbital (50 mg/kg, i.p.) and L4 and L5 DRGs were then removed. All experimental procedures were approved by the Institutional Animal Care and Use Committee at University of Texas medical Branch and were in accordance with the guidelines of the National Institutes of Health and of the International Association for the Study of Pain (IASP).

### Western blots

For Western Blot experiments, DRGs were lysed in 100 μl of RIPA buffer (1% NP-40, 0.5% Na dexycholate, 0.1% SDS, PMSF 10 μl/ml and aprotinin 30 μl/ml). The cell lysates were centrifuged at 10,000× g for 12 min at 4°C. The concentration of protein in homogenate was determined using a BCA reagent (Pierce). A 50 μg of total protein sample from each group was loaded onto a SDS-PAGE, transferred to a PVDF membrane, incubated with a blocking buffer (1 × TBS with 5% w/v fat free dry milk) for 2 hrs and then with rabbit anti-P2X3 (1:1000; Alomone), or anti-P2X7 (1:1000, Alomone) for another 2 hours at room temperature. The PVDF membrane was then washed 5 times with TBST (1 × TBS and 1% Tween 20), incubated with anti-rabbit peroxidase-conjugated secondary antibody (1:1000; Santa Cruz) for 1 hr at room temperature and washed 5 times with TBST. The immunoreactive proteins were detected by enhanced chemiluminescence (ECL kit; Amersham Biosciences) and visualized by exposing the PVDF membrane onto an x-ray film. Loading controls were determined by stripping the blots with a buffer containing 100 mM 2-mercaptoethanol, 62.5 mM Tris-HCl, and 2% SDS for 20 min and re-probing with mouse anti-Actin (1:1000, Chemicon) for 2 hr. After washing, the membrane was incubated with anti-mouse peroxidase-conjugated secondary antibody (1:1000, Chemica) for 1 h. The intensity of protein bands was determined using the NIH Image 1.63 software.

### Immunohistochemistry

To immunostain DRG cells, rats were deeply anesthetized with pentobarbital (80 mg/kg, i.p.) and fixed with 4% paraformaldehyde plus 0.2% picric acid. The L4 and L5 DRGs were removed, postfixed for 1 hr and put in a 30% sucrose solution overnight. Tissue was then embedded in an OCT compound and cut into 10 μm transverse DRG sections on a cryostat and washed with normal goat serum (10%) and Triton X (0.2%). Primary antibodies used included rabbit anti-P2X7R (Alomone, 1:200) or guinea pig anti-P2X3R (Chemicon, 1:10,000). Secondary antibodies included FITC or Texas Red anti-rabbit, or anti-guinea pig IgG (Vector Lab, 1:200). To compare the fluorescence intensity of labeled cells in control and test groups, tissue samples from different groups were processed simultaneously and analyzed using an identical fluorescence intensity threshold. To avoid the possibility of double-counting, labeled cells on every fifth DRG section were analyzed.

### Electrophysiology

To record the electrical activity of DRG neurons, one L4 or L5 DRG with an attached sciatic nerve was isolated from a rat. The preparation was superfused with the artificial cerebral spinal fluid (ACSF), which contained (in mM) 115 NaCl, 5.6 KCl, 1 NaH_2_PO_4_, 2.0 CaCl_2_, 1 MgCl_2_, 11 glucose, and 25 NaHCO_3_, at room temperature. Sharp electrodes filled with 3 M potassium acetate with a resistance ranging from 50 MΩ to 90 MΩ were used for recordings. DRG neurons were stimulated either by square pulses (0.1-1 ms) applied to the sciatic nerve through a suction electrode or by 40 ms current steps applied to the soma through an intracellular recording electrode. Only data obtained from cells with a resting potential ≤ - 40 mV and overshooting spikes were accepted for analyses. In adult DRG, cells were classified as Aβ, Aδ or C cells by axonal conduction velocity (CV) with the criteria of CV > 8.5 m/s as Aβ cells, CV = 1-8.5 m/s as Aδ cells, and CV < 1 m/s as C cells. In cases where CVs could not be established, the action potential duration (APD), i.e., duration measured at the half peak AP amplitude, and the existence of inflection on the falling phase of action potential (AP) were used as the criteria to separate cell groups [27]. Cells with an APD > 3.8 ms and AP inflection were considered as C cells; cells with an APD ≤ 3.8 ms and AP inflection were categorized as Aδ cells; cells with an APD < 3.8 ms and no AP inflection were considered Aβ cells. The same APD and AP inflection criteria were used to categorize cell types of DRGs in early postnatal ages, during which most of the axons were unmyelinated [28]. Only C and Aδ DRG neurons were included in our analyses. Chemicals, e.g., α,β-meATP (100 μM) and TNP-ATP (1 μM) were bath applied at a flow rate of ~ 4 ml/min. A membrane potential change of ≥ 2 mV, which was larger than 2 times the standard deviation of the baseline level, was set as a positive response, similar to the criterion used by Stebbing et al. [29].

### siRNAs

Small interfering RNA directed against P2X7 mRNA (P2X7R-siRNA) or a non-targeting control siRNA (con-siRNA) (20 μM, 10.5 μl) (Thermo Scientific) was used in our experiments. The sense sequence of con-siRNA was 5'-UAGCGACUAAACACAUCAA-3'. The P2X7R-siRNA consisted of four pooled 21-necleotide duplexes. The sequences were as follows: (1) Sense GAAACUGCCUCCCGUCUCAUU; Antisense 5'-P. UGAGACGGGAGGCAGUUUCUU; (2) Sense GGAUCCAGAGCGUGAAUUAUU; Antisense 5'-P UAAUUCACGCUCUGGAUCCUU; (3) Sense CCAAGCCGACGUUAAAGUAUU; Antisense 5'-P UACUUUAACGUCGGCUUGGUU; (4) Sense GAUACGCCAAGUACUAUAAUU; Antisense 5'-P UUAUAGUACUUGGCGUAUCUU. SiRNA-polyethyleneimine (PEI) complex (0.18 μl 100 mM PEI per μg RNA) was prepared according to the method described by Tan et al. [30] before use.

### Behavioral experiments

Nocifensive, i.e., flinching, behavioral studies were conducted on P15-16 and adult rats, which were treated with either con-siRNA or P2X7R-siRNA eight to nine days before. The siRNA was applied intrathecally using a modified direct transcutaneous intrathecal method [31]. Rats were placed under individual plexiglass domes and acclimated for 1 hr before experiments. Following an injection of α,β-meATP (1 mM, 50 μl), rats were immediately put back under the dome. The flinching behavior was assessed by determining paw withdrawal duration (PW duration), i.e., the total time that the hindpaw was lifted in the air in a 1 min time bin [32,33].

### Data analyses

Data were expressed as means ± SE or as percentages. Student's *t*, Fisher Exact or Mann-Whitney tests were used to access the significance of changes. Comparisons between multiple means were done with one-way analysis of variance (ANOVA) followed by the Holm-Sidak *post hoc *test. A *P <*0.05 was considered significant.

## Results

### A high percentage of DRG neurons express P2X3Rs in immature rats

**Figure 1:**
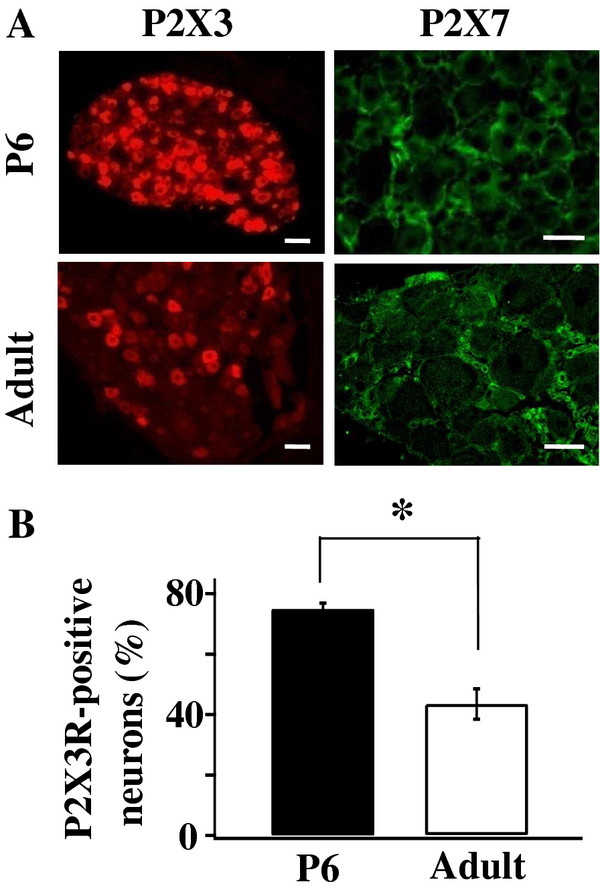
**P2X3Rs are expressed in a higher percentage of DRG neurons in immature rats**. (**A**) Immunocytochemical staining of P2X3Rs and P2X7Rs in DRGs of P6 and adult rats. P2X3R labels (red) were found only in neurons; P2X7R labels (green) were found only in SGCs. (Scale bars: P2X3R, P6 = 40 μm, Adult = 50 μm; P2X7R, P6 = 25 μm, Adult = 30 μm) (**B**) Percentage of DRG neurons expressing P2X3Rs decreases significantly during postnatal development (P6 rats: 74.83 ± 2.03%, n = 3; adult rats: 43.45 ± 5.03%, n = 3. Student t-test, *P < 0.05)

We first determined if P2X3Rs are expressed only in DRG neurons and P2X7Rs are expressed only in SGCs in immature rats as observed in adult cells [5]. DRG neurons and SGCs were differentiated by their morphological characteristics. The somata of DRG neurons had a rounded appearance with diameter ≥ 10 μm [34]. Each soma had a prominent nucleus with relative clear nucleoplasm and a low nucleus/cytoplasm ratio [35]. On the other hand, SGCs were elongated (width ≤ 2 μm) flattened cells that enveloped each neuronal soma [35,36]. The SGC had a flattened nucleus which contained dense chromatins and a high nucleus/cytoplasm ratio. In P6 rats, P2X3R immunostain was found only in DRG neurons, not in SGCs (Figure 1A). On the other hand, P2X7Rs were found only in SGCs (Figure 1A). An obvious difference in P2X3R distribution between immature and adult DRGs is that a high percentage of DRG neurons expressed P2X3Rs in immature rats. In P6 DRGs, 74.83 ± 2.03% (n = 3) of DRG neurons expressed P2X3Rs whereas only 43.45 ± 5.03% (n = 3) neurons expressed P2X3Rs in adult DRGs neurons (Figure 1B).

### The activity of P2X3Rs in DRG neurons decreases with rat age

**Figure 2:**
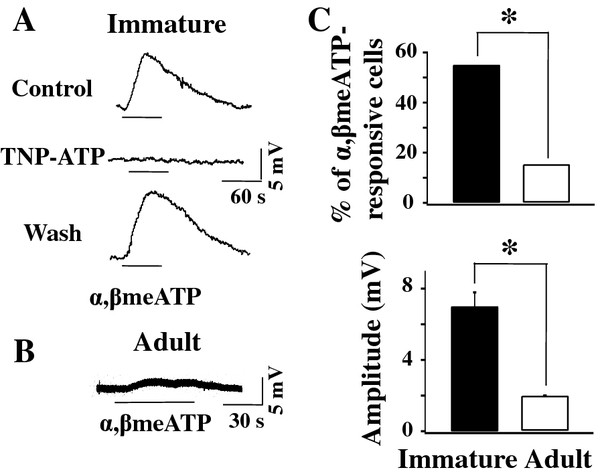
**High P2X3R-mediated activity in DRG neurons of immature rats**. (**A**) α,β-meATP induced significant depolarizations in DRG neurons of immature rats. In this example, α,β-meATP evoked 8.6 mV depolarization in a P4 DRG neuron. The depolarization was reversibly blocked by the P2X antagonist, TNP-ATP (1 μM). (**B**) α,β-meATP induced a much reduced, i.e., 2 mV, depolarization in an adult DRG neuron. (**C**) The percentage of DRG neurons responded to α,β-meATP was high in immature rats and greatly reduced in adult rats (P3-P14 rats: 55.0%, adult rats: 15.4%. Fisher exact test, *P < 0.05). The amplitude of depolarizations induced by α,β-meATP was reduced as rats matured (P3-P14 rats: 7.0 ± 0.8 mV, n = 11; adult rats: 2.0 ± 0 mV, n = 2. Mann-Whitney test, *P < 0.05)

We then determined the function of P2X3Rs in DRG neurons by measuring their responses to the P2XR agonist, α,β-meATP. DRG neurons were categorized in C, Aδ and Aβ cell groups using the criteria described in the METHODS section. Responses to α,β-meATP were studied only in C and Aδ cells. Sharp intracellular microelectrodes were used to measure the depolarization induced by bath applied α,β-meATP (100 μM). Under current clamp conditions, 11 out of 20 (55.0%) recorded DRG neurons from P3-P14 rats responded to α,β-meATP with ≥ 2 mV depolarization (Figure 2). The average amplitude of α,β-meATP induced depolarizations was 7.0 ± 0.8 mV (n = 11). In four immature neurons, the effect of the P2X1R and P2X3R antagonist, TNP-ATP, was also studied. TNP-ATP completely blocked the α,β-meATP-induced depolarization (Figure 2A). Only 2 out of 13 (15.4%) neurons tested responded to α,β-meATP in adult DRGs (Figure 2B). The average amplitude of depolarizations induced by α,β-meATP in adult DRG neurons (2.0 ± 0.0 mV, n = 2) was much smaller than that obtained in immature DRGs (Figure 2C).

### P2X7R-P2X3R inhibitory control exists in immature DRGs

**Figure 3:**
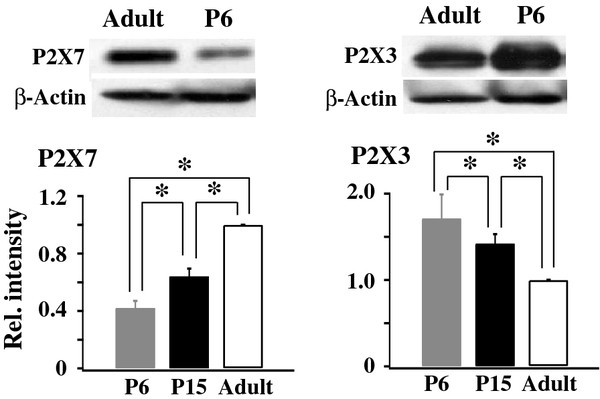
**P2X3R expression decreases and P2X7R expression increases with rat age**. Western blot analyses of P2X3Rs and P2X7Rs were conducted on the DRG protein samples isolated from P6, P15 and adult rats. P2X3R and P2X7R expressions were expressed in relative intensity with respect to those of adult rats, which was set at 1.0. During postnatal development, the P2X7R expression increased (P6/Adult: 0.42 ± 0.05; P15/adult 0.64 ± 0.06; n = 3-5), while the P2X3R expression decreased (P6/adult: 1.71 ± 0.28; P15/adult: 1.42 ± 0.11; n = 5) (One-way ANOVA, *P < 0.05)

In a previous study [5], we showed that reducing P2X7R expression or blocking P2X7R activity in SGC cells upregulates P2X3R expressions in adult DRG neurons. This P2X7R-P2X3R inhibitory control is important in maintaining proper functions of P2X3Rs. To determine if such P2X7R-P2X3R control is also present in immature DRGs and if it contributes to the enhanced P2X3R expression in immature rats, we first compared the P2X3R and P2X7R protein expressions in DRGs isolated from P6, P15 and adult rats. As rat aged, the P2X7R expression increased (P6/Adult: 0.42 ± 0.05, n = 5; P15/adult 0.64 ± 0.06; n = 3). At the same time, the P2X3R expression was found to decrease (P6/adult: 1.71 ± 0.28; P15/adult: 1.42 ± 0.11; n = 5) (Figure 3). Thus, P2X7R and P2X3R expressions are negatively correlated, similar to those observed in adult rats.

**Figure 4:**
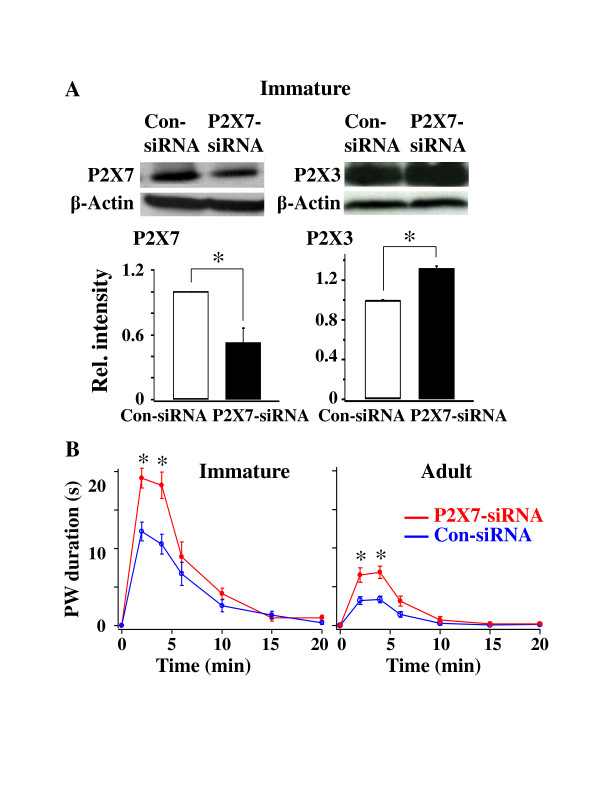
**P2X7R-P2X3R inhibitory control exists in immature rats**. (**A**) P2X7R-siRNA treatment down-regulated P2X7R expression while increased P2X3R expression in P15-16 rats. P2X3R and P2X7R expressions in P2X7R-siRNA rats were expressed in relative intensity with respect to those in Con-siRNA rats, which was set at 1.0. (**B**) The α,β-meATP induced much more pronounced P2X3R-mediated flinching responses in con-siRNA immature rats than in adult rats. In both immature and adult rats, α,β-meATP evoked significantly larger flinching responses in P2X7R-siRNA rats than in con-siRNA rats.

To determine the functional consequence of P2X7R-P2X3R expression control in immature rats, nocifensive, i.e., flinching, behavioral studies were conducted on P15-16 and adult rats, which were treated with either con-siRNA or P2X7R-siRNA. We applied P2X7R-siRNA intrathecally to P7 rats to down regulate P2X7R expression. Eight to nine days later, the effect of P2X7R-siRNA on P2X3R-mediated flinch behaviors induced by α,β-meATP application to the rat left hindpaw was determined. We first determined if P2X7R-siRNA is effective in knocking down P2X7R expression and if P2X3Rs are upregulated. Following P2X7R-siRNA treatment, P2X7R expression was reduced (P2X7R-siRNA/Con-siRNA = 0.52 ± 0.14, n = 4, Student t-test, P < 0.05), while the P2X3R expression in immature P2X7R-siRNA rats was increased (P2X7R-siRNA/Con-siRNA = 1.32 ± 0.02, n = 3, P < 0.05) (Figure 4A). In presence of con-siRNA, α,β-meATP-induced flinching responses were much more pronounced in immature rats than those found in adults. This result is consistent with the observations that both P2X3R expression and function are elevated in immature rats (Figures 1 and 2). Following P2X7R-siRNA treatment, the α,β-meATP-induced flinch behaviors in immature rats were significantly enhanced, similar to that observed in adult rats (n = 4-7, One-way ANOVA, P < 0.05) (Figure 4B). Thus, a lower expression of P2X7Rs in immature DRGs resulted in a higher expression and hence an enhanced function of P2X3Rs. The observation suggests that P2X7R-P2X3R inhibitory control plays an important role in regulating the expression and function of P2X3Rs in immature rats, as shown in adult rats (Figure 4B) [5].

## Discussion

Our immunocytochemical study showed that the percentage of DRG neurons expressing P2X3Rs decreases from 74.8% in P6 rats to 43.5% in adult rats (Figure 1), a result similar to those obtained in mice [23]. During postnatal development, P2X3R switches from being universally expressed in DRG neurons to limitedly expressed in IB4-positive neurons [23]. In adult DRG neurons, P2X3Rs are localized in small and medium-sized DRG neurons that process nociceptive signals [17,37,38]. To determine if the change in P2X3R expression corresponds to a change in P2X3R-mediated responses, we studied the depolarizations evoked by the P2X1R and P2X3R selective agonists, α,β-meATP, in intact DRG neurons. Since P2X1 expression in DRGs is low [38], the α,β-meATP-induced depolarization is likely to be mediated mainly by P2X3R-containing receptors in DRGs. We chose to determine P2X3R function in intact DRG neurons rather than in dissociated DRG neurons because the cell dissociation procedure appears to alter the percentage of cells responding to the agonist [29]. We found that 55% of recorded DRG neurons of P3-P14 rats responded to α,β-meATP (Figure 2). In contrast, α,β-meATP evoked depolarizations in only 15.4% of adult DRG neurons, a percentage similar to that obtained by Stebbing et al. [29]. Thus, an increased number of DRG neurons expressed P2X3Rs obtained from immunostain analyses is consistent with an increased number of neurons responding to α,β-meATP.

We also analyzed the α,β-meATP-induced depolarizations in individual DRG neurons. The slow kinetics of the α,β-meATP-induced depolarizations is likely a result of bath application of the agonist [29]. Because of the small percentage of adult DRG neurons responding to α,β-meATP, the kinetic difference in the depolarizing responses of immature and adult DRG neurons was not studied. We found that the amplitudes of α,β-meATP-induced depolarizations in individual adult DRG neurons is ~3.5 fold smaller than those recorded in immature neurons (Figure 2).

We further studied the P2X3R expression using Western analyses and P2X3R functions through behavioral analyses. In immature rats, the P2X3R protein expression was high (Figure 3) and the α,β-meATP application to the rat paw evoked much larger flinching behavioral responses in immature rats (Figure 4B). An increase in receptor expression in Western analyses and an increase in behavioral responses could result from a larger number of cells expressing receptors and responding to α,β-meATP respectively. They also can result from an increase in the receptor expression and function in individual neurons. Thus, an increase in the amplitude of α,β-meATP evoked depolarizations in individual neurons (Figure 2) is consistent with the results of Western and behavioral analyses. Combining immunocytochemical, electrophysiological and behavioral studies, we conclude that P2X3R expression and P2X3R-mediated cellular activity are high in immature DRG neurons and are reduced as rats mature. The high level of P2X3R expression in the somata of immature DRG neurons is likely to give rise to an enhanced transport of P2X3R receptor to their nerve terminals and thus increased α,β-meATP-induced behavioral responses. Nevertheless, the results do not exclude the possibility that developmental changes in the spinal circuitry and descending modulation could also contribute to the observed changes in the behavioral responses in immature rats.

We found that P2X3Rs are expressed only in DRG neurons and P2X7Rs are expressed only in SGCs (Figure 1) in P6 rats, similar to those found in adult DRGs [5]. There are similarities and differences between the distribution of P2X3Rs and P2X7Rs in DRGs and those in the developing rat brain. Similarities include that P2X3Rs are not found in glial cells [39] and P2X3Rs mRNA expression decreases while P2X7R expression increases and/or persists in the developing rat brain [25]. Different from DRGs, in addition to expressing in microglia cells, P2X7Rs are also expressed in brain neurons, e.g., Purkinje cells in the cerebellum [40].

As animals mature, the P2X7R expression increases as P2X3R expression decreases in DRGs (Figure 3). More important, we showed that knocking down P2X7R expression with P2X7R-siRNA significantly increases the P2X3R-mediated flinch responses in P15-16 rats (Figure 4). Thus, P2X7Rs negatively control the expression and function of P2X3Rs in immature rats just as those observed in adult rats [5]. Since P2X7R expression is low in P6-15 day rats (Figure 3), a reduced P2X7R-P2X3R inhibitory control is likely a mechanism contributing to the high expression and function of P2X3Rs in DRGs of immature rats.

Many challenges exist in pain management in infants. The expressions and functions of transmitters and receptors undergo changes during development [41]. The descending control pathways from the brainstem to the spinal cord in infants are not completely developed. Sensory inputs are integrated, both spatially and temporally [41]. In addition, verbal communication with infants is limited. Compared to adults, infants are more sensitive to repeated noxious stimuli and chronic tissue inflammation [42]. On the other hand, injury to peripheral nerves in infants gives rise to much less allodynia and neuropathic pain than those observed in adults [43,44]. Therefore, the information obtained from studies of pain treatment in adults is not directly applicable to infants. We showed before that, in adult rats, total P2X3R protein expression is upregulated in DRG neurons after inflammation [45] and the membrane trafficking of P2X3Rs increases following spared nerve injury [32]. It is of interest to determine if purinergic receptors respond to tissue and nerve injury in immature DRGs in a similar manner as in adult DRGs. Our observations that P2X3R and P2X7R expression are negatively correlated (Figure 3) and P2X7R-P2X3R inhibitory control also exists in developing rats (Figure 4) suggest that regulation of P2X7R expression in SGCs can be a useful strategy for controlling P2X3R activity in sensory neurons and relieving pain in infant patients.

## Competing interests

The authors declare that they have no competing interests.

## Authors' contributions

YC designed the study, performed Western blotting, immunocytochemical and electrophysiological experiments and prepared the manuscript. GL carried out the behavioral experiments. LMH designed and coordinated the study and prepared the manuscript. All authors read and approved the final manuscript.
